# Neuroblastoma and the epigenome

**DOI:** 10.1007/s10555-020-09946-y

**Published:** 2021-01-06

**Authors:** Irfete S. Fetahu, Sabine Taschner-Mandl

**Affiliations:** grid.416346.2 St. Anna Children’s Cancer Research Institute, Zimmermannplatz 10, 1090 Vienna, Austria

**Keywords:** Epigenetics, DNA methylation, Histone modifications, MicroRNAs, Chromatin remodeling, Neuroblastoma

## Abstract

Neuroblastoma (NB) is a pediatric cancer of the sympathetic nervous system and one of the most common solid tumors in infancy. Amplification of *MYCN*, copy number alterations, numerical and segmental chromosomal aberrations, mutations, and rearrangements on a handful of genes, such as *ALK*, *ATRX*, *TP53*, *RAS/MAPK* pathway genes, and *TERT*, are attributed as underlying causes that give rise to NB. However, the heterogeneous nature of the disease—along with the relative paucity of recurrent somatic mutations—reinforces the need to understand the interplay of genetic factors and epigenetic alterations in the context of NB. Epigenetic mechanisms tightly control gene expression, embryogenesis, imprinting, chromosomal stability, and tumorigenesis, thereby playing a pivotal role in physio- and pathological settings. The main epigenetic alterations include aberrant DNA methylation, disrupted patterns of posttranslational histone modifications, alterations in chromatin composition and/or architecture, and aberrant expression of non-coding RNAs. DNA methylation and demethylation are mediated by DNA methyltransferases (DNMTs) and ten-eleven translocation (TET) proteins, respectively, while histone modifications are coordinated by histone acetyltransferases and deacetylases (HATs, HDACs), and histone methyltransferases and demethylases (HMTs, HDMs). This article focuses predominately on the crosstalk between the epigenome and NB, and the implications it has on disease diagnosis and treatment.

## Introduction

### The biological basis of neuroblastoma and the importance of the epigenome to NB

Neuroblastoma is a developmental neoplasm of the autonomic nervous system that primarily affects young children [1–3]. A hallmark of NB is its heterogeneous clinical presentation, ranging from tumors that spontaneously regress (~ 50% of infants) to widely disseminated tumors that are frequently resistant to multimodal treatments like chemoradiotherapy or a combination of stem cell transplantation and immunotherapy in the other half of the subjects [4–7]. In the latter patient cohort, namely high-risk NB, the survival rate is < 40% [6, 8], highlighting the elusive nature of the disease. Cancer is caused by heritable aberrations in genes that regulate cell division, proliferation, and death [9–12]. Such critical genetic factors underlying NB onset and progression include amplification of *MYCN*, deletions of *TP53*, mutations or amplifications of *ALK*, rearrangements of *TERT*, deletions or mutations of *ATRX*, and segmental chromosomal aberrations [1, 13–19]. However, recent whole-genome sequencing studies have identified a scarcity of recurrent somatic alterations [13, 14, 20], which has hampered the efforts to develop targeted therapeutics for all NB patients. Therefore, the primary challenge in the identification and validation of diagnostic tools and treatment agents is the accurate representation of NB biology and diversity.

Malignancies generally involve both the genetic and epigenetic components that work in a concerted and multilayered fashion. Epigenetic mechanisms, such as DNA methylation, histone modifications, and non-coding RNAs, coordinate the maintenance of the epigenome [21, 22]. Together, they determine whether chromatin is transcriptionally permissive or repressive. Changes in this balance can, in turn, alter transcriptional programs that promote cancer progression by increasing cancer cell plasticity or by directly silencing tumor suppressor genes [12, 21, 23]. Several lines of evidence in various cancer models have demonstrated that aberrations in the epigenome largely occur by disrupting the epigenetic machinery and are strongly correlated with cancer progression [24]. Large-scale genome sequencing projects have shown that roughly 50% of human cancers harbor mutations in chromatin proteins, causing malignant cells to exhibit genome-wide alterations in DNA methylation, chromatin structure, and regulatory element activities [25–27]. These findings not only imply a causative role for epigenetics in tumorigenesis, but also help to identify potential therapeutic targets.

## Regulation of oncogenes and tumor suppressor genes by the epigenome

Epigenetic mechanisms are processes that have been associated with growth and development, and any dysregulations in this balance result in disease onset and progression. Aberrant gene expression as a result of the perturbed epigenome is a frequent event in cancer [21, 27]. DNA methylation primarily affects cytosines in the context of CpG dinucleotides, which is generally correlated with transcriptional repression [28, 29]. Other key chromatin remodeling activities include histone modifications, which undergo covalent posttranslational modifications (e.g., acetylation, methylation, and phosphorylation). While loss of histone acetylation has been established as a mark that is inversely correlated with gene expression, histone methylation can lead to either gene activation or silencing, depending on the degree of methylation (mono-, di-, or tri-methylation), type of amino acid residue impacted, and their location in the histone tails [30, 31]. Finally, microRNAs, which are short non-coding RNAs (17-22nt), negatively regulate gene expression, primarily by interacting with the 3′untranslated regions (UTR) of mRNAs [32]. Jointly, the abovementioned chromatin and gene regulatory mechanisms, and their role in oncogenesis in the context of NB will be the focus of this review. We will also discuss the current and emerging drug therapies that target these epigenetic regulators.

### Role of DNA methylation in gene expression in neuroblastoma

**Fig. 1:**
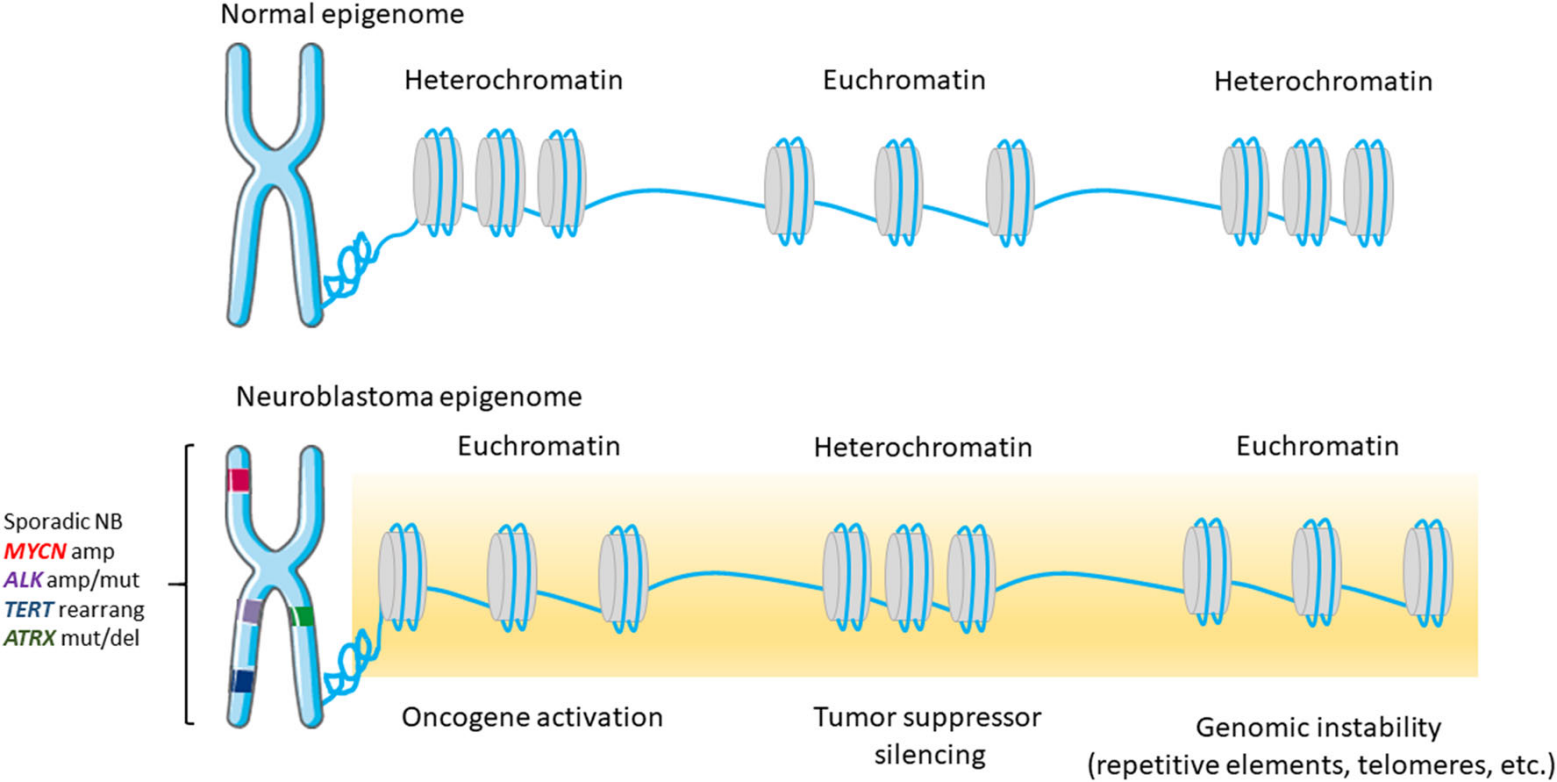
Proposed structural changes of chromatin in neuroblastoma cells. In normal cells, the chromatin structure is tightly regulated by DNA methylation and histone modifications, ensuring proper gene activation/silencing as well as genome integrity. The epigenome of NB cells is characterized by abnormally global open nucleosome configuration (euchromatin), interspersed with silenced genes (heterochromatin). Abbreviations: amp, amplification; mut, mutation; rearrang, rearrangement; del, deletion. Chromosome drawing was adapted from https://smart.servier.com/

DNA methylation at the fifth position of cytosine (5mC) in CpG dinucleotides is a major epigenetic mechanism involved in genome programming and reprogramming, and determination of cell fate during development, thus any disturbances in DNA methylation may give rise to disorders, such as cancer [21, 27]. Near gene promoters, transcription start sites, and/or first exons, CpG dinucleotides cluster in short DNA stretches, which are referred to as CpG islands (CGI), and most (80–95%) of them remain devoid of DNA methylation [33]. The majority of the CpG sites occur in intergenic regions and repetitive genomic sequences to maintain a transcriptionally inactive state [12]. Changes in the cancer epigenome are generally associated with loss of global DNA methylation and gain of DNA methylation at specific gene promoters. The consequences of loss of global methylation include chromosomal instability, loss of imprinting, and activation of transposable elements, thereby leading to disturbances in the genome [11, 22, 27] as exemplified in Fig. 1. Genome instability causes cancer genotypes to continuously change and evolve and can manifest itself genetically on several different levels, ranging from simple DNA sequence changes to structural and numerical abnormalities at the chromosomal level, all of which are observed in NB [34, 35].

Understanding the impact of normal and aberrant DNA methylation is one key area of interest, considering that drugs that result in DNA hypomethylation have already been approved by the US Food and Drugs Administration (FDA) and/or the European Medicines Agency (EMA) for the treatment of certain malignancies. DNA methylation is carried out by the DNA methyltransferases (DNMTs) [36]. During cell division, DNA methylation is established by DNMT1 or the maintenance DNA methyltransferase, while DNMT3A&3B are responsible for the *de novo* methylation. Co-transfection of Dnmt3a&3b in murine NB cell lines was associated with increased cisplatin resistance, while inhibition of these enzymes with the DNMT inhibitor, 5′-azacytidine (5-aza), resulted in an increased cisplatin response [37]. Treatment of mouse and human NB cell lines with another DNMT inhibitor, 5-aza-2′-deoxycytidine (DAC), led to reduced DNA methylation levels and inhibited DNA synthesis, cell proliferation, and colony-forming activity [38, 39]. Furthermore, treatment of NB cell lines with DAC and chemotherapeutic drugs, including cisplatin, doxorubicin, and etoposide, resulted in a superior therapeutic outcome compared to the treatments without DAC [40]. DAC, also known as decitabine has been approved for the treatment of several hematological malignancies. A phase I clinical trial showed that low doses of this drug in combination with cyclophosphamide have tolerable toxicity levels, however, the doses of DAC needed to produce a clinically relevant response in NB tumors are far higher than the physiologically tolerable levels [41]. Use of SGI-1027, a DNMT pan-inhibitor (DNMT1/DNMT3A/DNMT3B), and nanomycin A, a DNMT3B inhibitor in NB cell lines, resulted in a higher cytotoxicity when given alone or in combinatorial treatment with doxorubicin, and this effect was independent of the *MYCN* amplification status [42]. In ganglioneuroblastoma, the expression of DNMT3B7, which is a truncated isoform of DNMT3B, is higher than in NB patients, and this results in reduced cell growth and induced differentiation. Analysis of the transcriptome of the DNMT3B7 overexpressing cells showed upregulation of genes involved in the retinoic acid pathway, and treatment with all-trans retinoic acid-induced NB cell differentiation [43]. Together, these studies suggest that inhibition of DNMTs promotes a phenotype that is characterized by increased cell differentiation and reduced proliferation, however, it remains to be seen how this would translate clinically in patients. Currently, three clinical trials have completed phase I studies for decitabine in NB, and a fourth study is ongoing (phase II) for genistein (Table 1).

**Table 1:** Completed and ongoing clinical trials for DNMT and HDAC inhibitors in neuroblastoma (mentioned in this article)

Category	Drug name	Clinical trial phase	Clinical trial ID
DNMT inhibitors	Decitabine	Phase I	NCT01241162
		Phase I	NCT00075634
		Phase I	NCT03236857
	Genistein	Phase II	NCT02624388
HDAC inhibitors	Vorinostat	Phase I	NCT00217412
		Phase I	NCT01132911
		Phase I	NCT01019850
		Phase I	NCT01208454
		Phase I	NCT04308330
		Phase II	NCT02035137
		Phase II	NCT02559778

*DNMT*, DNA methyltransferase; *HDAC*, histone deacetylase

Several studies in recent decades have used DNA methylation to distinguish healthy cells from diseased cells. Studies using targeted bisulfite sequencing for candidate genes and the various array platforms have suggested dysregulated methylation in NB also. Loss of apoptotic features by cancer cells is a commonly observed event in tumorigenesis. A study focused on investigating gene-specific hypermethylation as an alternative mechanism for loss of caspase 8 (CASP8) expression in NB cases that otherwise do not display deletions on this gene. Using methylation-sensitive PCR, the authors showed that in NB cell lines, loss of CASP8 expression was associated with increased levels of DNA methylation. Treatment with the DNA-demethylating drug, 5-aza, restored the expression of CASP8 in 2/3 of investigated cell lines. Caspase 8 is involved in apoptosis, and loss of its expression, exclusively in *MYCN*-amplified NB tumors, allows for unhinged cell proliferation. The cell line data were validated in a NB patient cohort as well [44]. Other genes involved in regulation of apoptosis include the anti-apoptotic decoy receptors (DcRs), *DcR1* and *DcR2*. Methylation analyses in cell lines and NB patient samples showed dense methylation on these genes as a mechanism, which was linked to their loss of expression [45]. Interrogation of the methylation levels of 45 candidate genes in NB cell lines and tumors revealed, among others, another proapoptotic gene, *TMS1*, to be hypermethylated, and this was typically observed in stage 4 tumors [46]. Other studies analyzing the methylation levels of tumor suppressor genes showed loss of *RASSF1A* expression by promoter methylation [47, 48]. Additional studies focusing on nine specific tumor suppressor genes in cancer interrogated methylation levels at the CpG islands of these genes in NB patient samples. In addition to *RASSF1A*, the study identified *BLU* and *DKFZp451I127*, which were marked by aberrant methylation in NB. Methylation levels of *PCDHB* CGIs were used as a defining measure for the CpG island methylator phenotype (CIMP), a high-risk NB phenotype, and this was marked by poor overall survival (OS) in a Japanese cohort [49]. Similarly, another study showed that aberrant methylation of the *PCDHB* family members is associated with other clinical factors that indicate poor clinical outcome, such as *MYCN* amplification, metastatic tumor stage, and older age of the patient at the time of diagnosis [50]. The relevance of methylation in these CGIs was also tested in a German NB cohort, and the authors found even stronger correlation between CIMP-positive phenotype and poor OS as well as disease-free survival (DFS) compared to the CIMP-negative cases. Additionally, all cases in this cohort that displayed amplification of *MYCN* were marked as CIMP+ phenotype and affected OS and DFS independently of age and stage of disease [51].

Targeted DNA methylation analysis has identified several tumor suppressor genes, which are otherwise rarely mutated, to be regulated by methylation. Thus, study of DNA methylation status in NB has fueled the development of potential new screening strategies that could have a diagnostic and prognostic application. Earlier reports employing the Illumina 27k methylation array reported a set of 8 genes that were methylated in NB cell lines. Further investigations showed that 3 out of 8 genes (*SCNN1A*, *PRKCDBP*, and *KRT19*) were marked by differential methylation, which allowed to distinguish several subsets of individuals diagnosed with NB, where lower methylation levels were associated with favorable outcome in patients [52]. Another study using methyl-specific PCR along with methyl-CpG-binding domain (MBD) sequencing identified 43 genes that were marked by differences in DNA methylation in NB cell lines after treatment with DAC and primary NB samples. Validation studies in patient samples showed that the methylation levels of 2/43 genes (*HIST1H3C* and *GNAS*) were associated with OS and/or event-free survival (EFS) [53]. The same group proposed a panel of 58 gene signatures in a larger patient cohort (*n* = 396), suggesting that methylation levels of these genes allow for accurate OS and EFS prediction [54]. However, it is unclear why the genes identified in their first study were not present in the latter study, given that the same experimental approaches were utilized. Finally, a study performed in 105 NB patients determined DNA methylation by Illumina 450k methylation array showed distinct clusters that were separated based on methylation levels, age, and *MYCN* amplification status. Multivariate survival analysis showed distinct survival outcomes, where the groups that were marked by higher methylation levels were linked to poor outcome. However, this group was characterized by amplified *MYCN* or higher levels of MYCN [50], therefore, it is unclear to which extent the perturbed DNA methylation levels alone contribute to the outcome prediction.

**Fig. 2:**
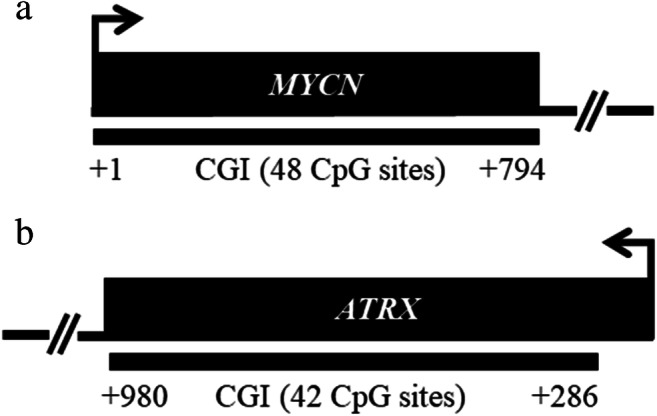
Identification of CpG islands (CGI) on *MYCN* and *ATRX* genes. Location of CGIs along with the number of CpG sites is indicated by black bars and was set relative to gene start (Ensemble Genome Browser, GRCh38). Only promoter and first exon regions were considered for the CGI identification. Arrows denote promoter orientation

DNA methylation is critical during development, and with NB being a neurodevelopmental tumor, it is pivotal to understand whether deranged methylation is linked to any of the specific developmental phases, thus providing more mechanistic insights into the disease etiology. A study of stage 4S NB, which generally undergoes spontaneous regression and is associated with excellent prognosis, aimed to understand the methylome of this specific subtype of NB. Using the MBD sequencing approach, the authors compared the methylation status between samples in stages 4S and 1, 2, and 4. None of the samples displayed *MYCN* amplification. They observed hypermethylation in genes associated with subtelomeric regions. Moreover, the study showed hypermethylation of genes that are involved with neural crest development and differentiation in 4S subset compared to the tumors in stages 1, 2, or 4 [55]. Another critical gene involved in telomere maintenance is telomerase reverse transcriptase (*TERT*). Expression of this gene is upregulated in high-risk NB, and this was associated with, among others, altered DNA methylation levels, which were also linked to poor clinical outcome [56]. ALK tyrosine kinase receptor (*ALK*) is another key gene in NB development, and several activating mutations have been reported in NB, and this was associated with unfavorable clinical outcome [57]. A study by Gómez et al. reported non-CpG methylation on the gene body of *ALK*, which was present in favorable NB tumors, but was otherwise absent in aggressive NB tumors. Furthermore, the authors suggest that post-chemotherapy, the non-CpG methylation in unfavorable NB, was restored along with the reduced expression levels of ALK [58]. However, non-CpG methylation was determined using the Illumina 450k methylation array, which covers relatively few non-CpG sites, and that bisulfite sequencing cannot discriminate between the 5mC and 5hmC modifications. Considering that non-CpG methylation and hydroxymethylation (5mC&5hmC) are prevalent in pluripotent stem cells as well as in neurodevelopment [59], it is pivotal to study the contribution of these modifications individually. In addition, for instance, the methylation status of *ATRX* and *MYCN*, key genes in NB pathology, is not known, albeit both contain a CpG island (Fig. 2).

DNA methylation is a reversible process, which is mediated by the TET proteins, where 5mC can be oxidized in an iterative manner to 5-hydroxymethyl (5hmC)-, 5-formyl (5fC), and 5-carboxyl-cytosine (5caC) [60, 61]. However, the roles of oxidized derivatives of 5mC have not been studied in NB. Moreover, most of the proposed genes for screening are dependent on *MYCN* amplification status, which on its own is sufficient to provide an accurate NB diagnosis. How the NB onset and progression is associated with predictable altered genome-wide levels of 5mC and its oxidized forms at base-pair resolution remains to be investigated.

### Role of histone modifications and chromatin remodeling in gene expression in neuroblastoma

Chromatin structure and organization dictate gene activity throughout the genome. Posttranslational modifications of histone tails determine the chromatin configuration, enabling it to attain a more relaxed or condensed structure, which in turn allows for genes to become activated or silenced, respectively. The C- and N-terminal residues protruding from the nucleosomes undergo several posttranslational modifications, including acetylation, methylation, phosphorylation, and sumoylation, which primarily occur in lysine, arginine, serine, and/or threonine residues [31]. These covalent histone modifications are established and removed in a dynamic fashion by enzymes known as “writers” and “erasers,” allowing for chromatin plasticity.

Acetylation of lysine residues in the histones is mediated by histone acetyltransferases (HATs) and is generally associated with active gene transcription. Current evidence regarding the role of HATs in NB is limited, however, use of the synthetic compound BF1, a HAT inhibitor, in NB cell lines caused hypoacetylation of histone 3 (H3) and of lysine (K) 18 on H3 (H3K18), which was also marked by reduced cell growth [62]. Similarly, use of HAT inhibitors, PU139 and PU141, led to diminished lysine acetylation levels accompanied by reduced cell growth, both *in vitro* and *in vivo*. In addition, PU139 showed synergism with doxorubicin by reducing tumor growth [63, 64].

Histone acetylation is not a static entity, and as such the effects of HATs are antagonized by the catalytic activity of histone deacetylases (HDACs) [65]. In NB, the role of HDACs has been described by shedding light on the potential mechanisms involved in NB tumorigenesis, serving as potential markers aiding in patient stratification, as well as selection of novel therapeutic options in addition to the conventional treatment therapies. Studies showed that the following HDACs: HDAC2, SIRT1, and SIRT2 were upregulated by MYCN, which in turn promote stability and expression of MYCN protein, thus describing a positive feedback loop [66–68]. Inhibition of SIRT1 led to decreased tumorigenesis in mice [66]. Expression of several HDACs has been suggested as a potential approach for stratification of NB patients. Studies by Oehme et al. showed that the expression of HDAC8 and HDAC10 correlated with poor OS and EFS in NB [69, 70]. Pharmacological inhibition of HDAC8 *in vitro* and *in vivo* resulted in inhibition of proliferation and induction of differentiation [69, 71]. Additionally, levels of HDAC8 and HDAC10 anticorrelated with sensitivity to doxorubicin treatment in NB [70, 72]. Finally, kinome-wide RNAi screening revealed *ALK* as a candidate gene that gives rise to synthetic lethality in combination with HDAC8 inhibitors. Combined chemical inhibition of ALK and HDAC8 resulted in cell death or reduced cell viability in various cell lines with wild type *ALK*, or carrying amplified or activating mutations of *ALK*, respectively [73]. In recent years, several HDAC inhibitors have been included as a new category of anticancer treatment. Many ongoing clinical trials have included inhibitors of HDACs as a potential new line or as combinatorial treatment in NB. Treatment of NB cell lines with vorinostat (also known as suberanilohydroxamic acid, SAHA) resulted in growth arrest and promoted apoptotic pathways [74, 75], including in the cell lines that carry *MYCN* amplification [76]. In addition, vorinostat enhances the anti-tumor properties of various compounds, including flavopiridol and fenretinide [77, 78]. It also works in concert with conventional treatments, like chemotherapy (actinomycin D, paclitaxel) or radiotherapy [79–82]. Phase II clinical trials for the use of vorinostat in NB patients at the time of the submission of this review are still ongoing (Table 1). A report by Waldeck et al. showed that treatment of TH-MYCN transgenic mice for 9 continuous weeks with the pan-inhibitor of HDACs, panobinostat, increased survival rates compared to mice that were treated for 3 weeks only [83]. The combined treatment of high-risk NB cell lines with panobinostat and cisplatin, doxorubicin, or etoposide had synergistic effects in induction of apoptosis [84]. Finally, use of romidepsin in NB cell lines was also associated with reduced cell growth and increased cell apoptosis. This was evident in *MYCN*-amplified cell lines, p53 wild type or mutant cell lines, as well as those carrying *ALK* mutations [85, 86]. Growth inhibition by romidepsin was also evident in an immunocompromised mouse model [85, 86]. It remains to be seen how these drugs perform in clinical trials and whether they will be as efficient as in solid tumors or there will be a need to design more specific inhibitors.

Histone methylation can lead to either gene activation or repression, depending on the histone site that is methylated, the degree of methylation (e.g., mono-methylation, di-methylation, or tri-methylation), amino acid residues affected, and their position in the histone tail [31]. Histone methylation primarily occurs on the side chains of arginine (R), histidine (H), and lysine (K) residues and is a dynamic process mediated by histone methyltransferases (HMTs) and histone demethylases (HDMs). One of the most prominent HMTs, EZH2, a member of the polycomb repressive complex 2 (PRC2), which is responsible for establishing mono-, di-, and tri-methylation of H3K27, a marker of repressed chromatin (Table 2), is characterized by increased expression levels in NB [88]. Several studies reported that in *MYCN*-amplified NBs, the promoter of *EZH2* is occupied by MYCN, both *in vitro* and *in vivo*, thereby driving its expression [89–91]. Another mechanism linked to elevated levels of EZH2 in NB was attributed to regional gains encompassing *EZH2* gene in a NB patient [92]. Furthermore, studies in NB cell lines and patients showed that increased activity of PRC2 in *MYCN*-amplified tumors led to chromatin compaction, thus facilitating the silencing of gene networks that were marked by concomitant DNA hypermethylation [50]. Overexpression of EZH2 promoted an undifferentiated NB tumor phenotype and was associated with poor clinical outcome [93], while its pharmacological inhibition (tazemetostat) resulted in reduction of proliferation [94]. In addition, genetic silencing of *EZH2* was marked by increased cell differentiation, which was mediated by NTRK1 [93]. Another histone methyltransferase that has been described in NB is EHMT2, which carries out mono- and di-methylation of H3K9, a mark associated with heterochromatin. Chemical inhibition of EHMT2 by BIX-01294 resulted in reduced cell proliferation and induced apoptosis [95]. Another study showed that inhibition of EHMT2 led to re-expression of several tumor suppressor genes, such as *CLU*, *FLCN*, *AMHR2*, and *AKR1C1-3* [96]. Methylation on H3K79, a mark of permissive chromatin, is mediated by DOT1L. Inhibition of DOT1L using small molecule inhibitor SGC0946 led to lower methylation levels of H3K79, which was followed by reduced proliferation in cell lines carrying *MYCN* amplification, which also regulated DOT1L expression. Genetic ablation of DOT1L *in vivo* also reduced tumor growth and improved OS [97].

**Table 2:** A simplified list of the members of histone demethylases and methyltransferases (mentioned in this article)

Class of histone demethylases/methyltransferases	Histone demethylase /methyltransferase family	Histone demethylase /methyltransferase	Histone substrate	Gene expression
Erasers				
LSD demethylases	KDM1	KDM1A*	H3K4me2/me1	Repression
JMJC demethylases	KDM3	KDM3A	H3K9me2/me1	Activation
	KDM4	KDM4B	H3K9me3/me2	Activation
	KDM5	KDM5B	H3K4me3/me2/me1	Repression
	KDM6	KDM6A	H3K27me3/me2	Activation
		KDM6B	H3K27me3/me2	Activation
Writers				
EZH2	EZH2	EZH2	H3K27me3/me2/me1	Repression
EHMT2	EHMT2	EHMT2	H3K9me2/me1	Repression
DOT1L	DOT1L	DOT1L	H3K79me3/me2/me1	Activation

*H* histone, *K* lysine, *me1* mono-methylation, *me2* di-methylation, *me3* tri-methylation, *LSD* lysine-specific demethylase, *JMJC* Jumonji C domain-containing protein demethylase, *EZH2* enhancer of zeste 2 polycomb repressive complex 2 subunit, *EHMT2* euchromatic histone lysine methyltransferase 2, *DOT1L* DOT1-like histone lysine methyltransferase

*In interaction with androgen receptor, KDM1A changes substrate preference to H3K9me2/me1, which is associated with gene expression activation [87]

Earliest research on the role of histone demethylation in NB showed overexpression of LSD1 (KDM1A), a histone demethylase that specifically demethylates H3K4me2/me1 to unmethylated H3K4, which is associated with gene silencing. This resulted in a phenotype, which was characterized by poorly differentiated cells and was associated with poor prognosis. Pharmacological inhibition of LSD1 resulted in inhibition of tumor growth in a mouse model [98]. In addition, LSD1 co-localizes with MYCN at the promoters of *CDKN1A*, *CLU*, and *NDRG1* resulting in their repression of expression, where inhibition of LSD1 restored their expression, while combined inhibition of MYCN and LSD1 resulted in reduced cell proliferation and/or invasiveness in *MYCN*-amplified cell lines [99, 100]. Another critical process in tumorigenesis that is governed by LSD1 is autophagy by regulating the expression of mTORC1 through SESN2 [101]. What contributes to the high levels of LSD1 in NB tumors is not well known, however, studies reported miR-137 and miR-329 as regulators of LSD1 expression [102, 103]. Downregulation of miR-137 expression was linked to poor patient prognosis, and functional studies demonstrated that LSD1 is a direct target of miR-137 in NB cell lines [103]. The histone demethylase KDM3A, which removes me2/me1 from H3K9, was shown to be upregulated by MYCN in NB cell lines. This in turn led to increased proliferation by upregulating the expression of MALAT1 [104]. Expression profiling data showed that KDM4B, which catalyzes demethylation of H3K9me3/me2, a repressive chromatin mark, was upregulated in NB patients compared to ganglioneuroma or ganglioneuroblastoma. MYCN was shown to interact with and recruit KDM4B, and both genes could stratify a subgroup of patients that had poor clinical outcome [105]. The histone demethylase KDM5B, which targets H3K4me3/me2/me1, is upregulated in NB, and this confers the tumor cells with stem cell-like behavior and drug resistance. Paradoxically, the expression of KDM5B is negatively regulated by MYCN, and loss of expression of KDM5B resulted in reduced cell proliferation *via* Notch/Jagged signaling [106, 107]. Treatment of various NB cell lines with a small molecule inhibitor (GSK-J4) of KDM6A and KDM6B, demethylases of H3K27me3/me2 led to induced differentiation and apoptosis, as well as reduced proliferation in these cell lines. Moreover, GSK-J4 also effectively reduced the growth of tumors in patient-derived xenograft mouse models [108]. Similarly, another study showed that KDM6B promotes cell differentiation in NB, acting downstream of retinoic acid-HOXC9 axis. Expression of KDM6B is downregulated in high-risk NB, while high expression of KDM6B is a prognostic marker for better patient outcome [109]. The precise mechanisms by which the loss or gain of expression of these enzymes contributes to oncogenesis are yet to be fully investigated, especially since the inhibition of enzymes that promote permissive or repressive chromatin states both appear to contribute to reduced tumorigenic features of NB cells.

### Role of non-coding RNAs in gene expression

MicroRNAs (miRNAs) are a family of small non-coding RNAs, which regulate an array of processes, and have been shown to be aberrantly regulated in cancers. They negatively regulate gene expression, either by perfectly matching the mRNA transcript, which results in degradation or by imperfect matching that results in prevention of translation [110]. The studies about the role of miRNAs and other non-coding RNAs in NB development and therapy resistance, as well as their expression regulation by MYCN, are too numerous to comprehensively describe in this review and have been extensively reviewed in these articles [111–115]. MiRNAs are relatively stable and can be secreted and circulate in the blood, which makes them excellent biomarker candidates [116], which will also be the prime focus of this review section. Currently, there are ~ 2300 human miRNAs identified [117]. A study by Bray et al. in a cohort of 145 NB patients with different genetic backgrounds studied the expression of 430 miRNAs. The study reported aberrant expression of 37 miRNAs in *MYCN*-amplified tumors compared to the ones carrying one copy of *MYCN*. The authors further showed that a set of 15 miRNAs was predictive of OS with a specificity of 86.5% and a sensitivity of 72.7% [118]. Another study using the same approach (analysis of 430 loci by stem-loop RT-qPCR) in 69 NB patients calculated EFS by means of support vector machines (SVM) and actual survival times with Cox regression-based models (CASPAR), which were highly predictable and accurate (SVM-EFS accuracy: 88.7% and CASPAR-EFS probability: 0.19%) and were validated in an independent cohort (SVM-EFS accuracy: 94.74% and CASPAR-EFS probability: 0.25%). Here, too, *MYCN* amplification correlated with the deregulated expression of miRNAs. Moreover, 37 miRNAs identified in this study correlated as well with TrkA expression, which is a marker of favorable clinical outcome. Expression of the most significant TrkA-correlated miRNA, miR-542-5p, also discriminated between local and metastatic disease, and was inversely correlated with *MYCN* amplification and EFS [119]. Again, using the same approach by screening for the expression of 430 mature miRNAs, a study in a large NB patient cohort (*n* = 534) showed that a set of 25 miRNAs was an independent predictor of patient survival, which was able to discriminate the test patients with regard to progression free and OS in both high-risk NB patients and general NB population [120]. In the three abovementioned studies, there was an overlap of 16 miRNAs in the first two studies, and an overlap of 2 miRNAs (miR-190, miR-488) between all the three studies, indicating a potential use for diagnosis and prognosis in NB. Using next generation sequencing, a study conducted in 128 NB patients reported a set of 23 miRNAs that were marked by deregulated expression in the tumors, regardless of *MYCN* amplification status compared to the controls. Target genes of these miRNAs were involved with various cancer pathways, such as DNA repair, apoptosis, and FGFR/EGFR signaling [121]. A study performed in 30 NB patients, out of which 11 were characterized as high-risk NB, while the rest as low to intermediate risk, screened for 754 miRNAs. The authors identified a set of 38 differentially expressed miRNAs between these two groups of patients [122]. A screening of 851 miRNAs in a discovery cohort of 13 patients identified a set of 17 miRNAs that were able to stratify low-risk from high-risk NB patients. In a validation cohort of 214 NB patients, 15/17 miRNAs were again able to discriminate the two different risk groups. Furthermore, miR-487b and miR-410 were marked by loss of expression in the high-risk group and were associated with DFS in non-*MYCN*-amplified tumors, whereas miR-487b expression was associated with OS and DFS, independent of the clinical covariates [123].

Use of serum to perform screening of the miRNome identified 36 upregulated and 46 downregulated miRNAs in murine models with high-risk NB [124]. In a cohort of 8 NB samples and 20 controls, using serum isolated RNA, Murray et al. identified a panel of 5 miRNAs (miR-124, miR-9, miR-218, miR-490, and miR-1538), which were overexpressed in *MYCN*-amplified NB subjects compared to controls [125]. A comprehensive study in 185 NB patients screened 743 miRNAs in serum, where levels of a set of 9 miRNAs (miR-375, miR-124-3p, miR-323a-3p, miR-129-5p, miR-218-5p, miR-490-5p, miR149-5p, miR-873-3p, and miR-10b-3p) were a feature of metastatic tumors compared to patients who only had localized primary tumors. The levels of these miRNAs were higher in serum of mouse NB xenografts, and these levels also correlated with the tumor volume [126]. Finally, a study tested a set of miRNAs associated with exosomes in the serum of 52 NB patients to potentially identify a signature that could discriminate between patients that have a favorable or poor response to chemotherapy. A set of 3 exosomal miRNAs (let-7b, miR-29c, and miR-342) could stratify patients that had a good response compared to the patients that were characterized by poor response to chemotherapy [127].

Although most of these studies have provided limited mechanistic insight and mainly present the connection between miRNAs and NB in a purely correlative manner, they have begun to shed light on the miRNome abnormities in NB tumors and elucidate how global sequencing platforms provide new means that can be leveraged in NB diagnosis and prognosis.

## Regulation of the epigenome by core NB genes

Genetic lesions in chromatin-modifying enzymes and the consequent changes in the epigenetic landscapes have a direct impact on transcriptional programs that are fundamental to tumorigenesis [24–27]. Chromatin homeostasis is largely dependent on the interplay between the polycomb repressive complexes 1 and 2 (PRC), trithorax-group proteins (e.g., SWItch/Sucrose Non-Fermentable, SWI/SNF), and nucleosome remodelers [128]. Altered expression or mutations in constituents of these complexes disrupt this homeostasis. The PRC2 complex is responsible for trimethylated lysine 27 of histone 3, resulting in a shift towards a repressive chromatin state [27]. Overexpression of EZH2, which is the catalytic unit of PRC2, was reported in *MYCN-*amplified NB [50, 89, 91, 129], potentially silencing genes or networks of genes with a tumor suppressive role. The SWI/SNF complex opposes the effects of PRC2. Members of the SWI/SNF complex, most notably ARID1A/B, are among the most frequently mutated targets in all human cancers [130], including NB, resulting in a poor clinical outcome [20]. Alterations on ATRX, which codes for a SWI/SNF-like protein, have been reported in NB of children and adolescents, and are associated with overall poor survival and lack of appropriate treatments [131]. While the genetic role and to some extent also the epigenetic role of MYCN in NB are understood, the nature of epigenetic mechanisms involved in other types of NB, which encompass the majority of the NB cases, remains elusive.

### Epigenetic regulation by MYCN in neuroblastoma

The pathological activation of MYCN is a typical feature of highly aggressive and relapsed NB tumors, which are characterized by a poor survival outcome and lack of treatments [132, 133]. Downregulation of MYCN expression induces apoptosis and differentiation, reverses tumor stem-like features, and accompanies senescence in *MYCN*-amplified NB cells. Pharmacological targeting of MYCN, albeit so far, only indirectly, is defined by reduced tumor growth *in vitro* and *in vivo* [134–143]. The role of MYCN as a master transcription regulator in controlling cell proliferation and survival is well-established [144–148]. In addition to the classical gene expression regulation, MYCN has been reported to regulate various epigenetic processes, such as histone modifying enzymes and miRNAs, which are described in Sections 2.2 and 2.3 of this review [149, 150]. MYCN along with chromatin-modifying proteins has a strong influence on disease progression and metastasis, which makes all of these molecules key targets for therapy. However, considering their expression in a broad range of healthy cells and their global contribution in gene regulation activity, it is pivotal to develop a better understating that would lead to generation of drugs that produce specific effects directly influencing oncogenesis.

Chromatin-modifying proteins are attractive as therapeutic targets for cancer since their aberrant expression has been reported in various tumor types [21, 31, 151]. Recent studies have started to emerge and have garnered attention about the role of MYCN and super-enhancers (SE). Gene regulatory circuitries consisting of master transcription factors (TFs) carefully control cell fate and identity by governing the expression of a well-defined set of genes. These types of TFs are generally recruited by a SE, which in turn is controlled by a master TF, thus regulating expression of cell type-specific developmental gene programs [152]. Such core regulatory circuitries (CRCs) have recently been described in NB, which were implicated in two phenotypically divergent cell states: committed ((nor)adrenergic)) and uncommitted (neural crest cell/mesenchymal cell-like type of cells), where each type of cell identity was well-defined by CRC models, including ASCL1, EYA1, PHOX2B, HAND1&2, GATA3, SIX3, and AP-1 and MEOX1, MEOX2, SIX1, SIX4, SOX9, SMAD3 and WWTR1, PRRX1, respectively [153, 154]. Additionally, in *MYCN*-amplified cell lines, another set of CRCs was identified, which included MYCN, HAND2, ISL1, PHOX2B, GATA3, and TBX2. Treatment with inhibitors of bromodomain and extra-terminal (BET) proteins, JQ1 and cyclin-dependent kinase 7 (CDK7)/SE, TZH1 resulted in a significant expression downregulation of genes that were associated with these CRCs [155, 156]. Other studies have shown that use of TZH1 resulted in downregulation of MYCN, followed by a significant suppression of MYCN-dependent gene transcriptional amplification. *In vivo* studies demonstrated tumor regression in a mouse model without significant systemic toxicity. The striking selective inhibitory effects of TZH1 in *MYCN-*amplified cells may be a result of downregulation of SE-associated genes or gene networks, including MYCN [157]. In addition, combinatorial treatment of TZH1 with ponatinib or lapatinib, which inhibits, among others, the protein kinase phosphatase 1 nuclear targeting subunit (PNUTS) that interacts with MYC protein and suppresses its degradation, resulted in a synergistically induced NB cell apoptosis, while having little effect on normal cells. Moreover, genetic ablation of *PNUTS* resulted in decreased MYCN protein, but not RNA expression. This was associated with reduced cell proliferation and cell survival, indicating another potential drug for use as an inhibitor of MYCN [158].

A new avenue of promising cancer therapeutics are the BET protein inhibitors, which have shown great efficacy in several malignancies, where a common target in all of these studies was downregulation of MYCN [159–161]. Treatment with the BET-bromodomain inhibitor, JQ1, resulted in reduced proliferation, induced apoptosis, and differentiation [141, 162–164]. This also was characterized by increased survival rates *in vivo* in three different NB models [141]. In NB, low expression of natural killer (NK) cell-activating receptors is inversely correlated with the expression of MYCN. However, treatment with JQ1 resulted in more resistant NB cell lines to NK cell-activating receptors, which—along with the inhibition of MYCN—impaired also the function of c-MYC and p53 that regulate the expression of specific ligands (ULBP1-3) for NKG2D-activating receptor [165]. Finally, as with other lines of treatment for malignancies, drug resistance is a major hurdle. A recent study pointed out that PI3K pathway promotes resistance to BET protein inhibitors, and as such, inhibitors of PI3K along with BET protein inhibitors were suggested as an upfront combination to circumvent this drug resistance [166]. Mechanisms of action for the BET protein inhibitors are currently being elucidated, and it appears that they target and regulate a selective group of genes, where expression downregulation seems to be mediated through inhibition of transcription elongation as a primary mode of action.

### Chromatin remodeling in neuroblastoma by ATRX and ARID1A/1B

In the past decade, several major breakthroughs focusing on the identification of novel epigenetic enzymes that participate in epigenetic regulation of normal biological and disease processes have emerged. One such protein is ATRX, which when mutated leads to α-thalassemia, mental retardation, and X-linked (ATRX) syndrome. The *ATRX* gene encodes for a SWI/SNF-like protein, which plays diverse roles in chromatin remodeling [167, 168]. The SWI/SNF family of proteins restructures the nucleosome by regulating DNA-histone interactions, which change the conformation of the nucleosome, thus facilitating chromatin remodeling during transcription, DNA replication, and repair [169, 170]. Chromatin remodeling by ATRX is mediated by two of its highly conserved domains: (1) the N-terminal ATRX-Dnmt3-Dnmt3L (ADD) domain, which contains a GATA-like zinc finger, a PHD finger, and an alpha helix and (2) the ATPase/helicase domain that is located at the C-terminus. The ADD domain contains H3K9me3 binding pockets, and its binding is promoted by H3K9me3 and unmethylated H3K4, but is disrupted in the presence of H3K4me2/3 [171–173]. The ATP domain has DNA translocase and mononucleosome modulating pattern activities [174, 175]. Mutations in either domain are characterized by significant developmental delays and severe intellectual disabilities, among others [167, 176, 177]. The roles of *ATRX* mutations in pediatric and adult malignancies are emerging [178]. Our studies in a cohort of ~ 200 patients show that early disease progression and relapse are linked to, among others, intragenic deletions of the *ATRX* gene [179]. Similarly, others have reported alterations on the *ATRX* in NB of adolescents and young adults, which were associated with poor clinical outcome [131]. Additionally, *ATRX* mutation frequencies are prevalent in patients with INSS stage 4 disease and in the high-risk subgroup [180].

Mutations on *ATRX* and *MYCN* amplification are mutually exclusive [13, 180, 181]. ATRX and its interacting partner, DAXX, were shown to deposit histone variant H3.3 at heterochromatic regions (e.g., repetitive DNA, telomeres, etc.) [182–184]. NB specimens carrying mutations on ATRX or DAXX display an alternative lengthening of telomere phenotype [185]. However, recent evidence suggests that loss of ATRX-DAXX interaction is not critical in NB pathology [94]. Interestingly, DAXX mutations have not been reported in patients with ATRX syndrome [177], indicating potential tissue-specific interactions with other proteins. Moreover, mutations that generate in-frame fusion ATRX protein appear to change preference from repressive promoters to active promoters, thus regulating expression of neuronal genes through REST [94]. Another interacting partner of ATRX is MRN (Mre11, Rad50, and Nbs1), which was shown in HeLa and U2OS cell lines [186, 187], but whether this interaction persists in NB or not, and the role it plays, is not yet elucidated. Finally, ATRX contains a putative EZH2 interacting domain, which was identified through a yeast two-hybrid screen [188], but this has not yet been shown in mammalian cells. Recent studies show that NB cells are sensitive to EZH2 inhibitors, resulting in reduced cell growth [89, 94]. Whether this is mediated by interacting with ATRX or follows a different mechanism of action is not known. Thus, it is pivotal to identify and dissect the interacting partners of the ATRX complex, which will then allow proposing existing and new compounds for therapeutic use in NB.

Other members of the SWI/SNF complex, most notably ARID1A/B, are among the most frequently mutated targets in all human cancers [20, 130, 189, 190]. Mutations of these genes have also been reported in NB and are associated with treatment failure and poor survival rates [20, 191]. Similar to other SWI/SNF proteins, ARID1A&1B act to remodel chromatin and can have both activating and repressing effects on gene expression [192]. Loss of ARID1A expression in NB cell line, SK-N-SH, promoted cell invasion. In addition, this was associated with higher expression of matrix-metalloproteinases 2 and 9 (MMP2&9), along with increased expression of N-cadherin and nuclear translocation of β-catenin, whereas expression of E-cadherin was reduced [193]. Evidence from NB cell lines showed that treatment with 13-cis retinoic acid resulted in increased expression of ARID1A, which in turn reduced the expression of TERT. Promoter occupancy studies showed that *ARID1A* through SIN3A suppressed the expression of TERT, thus resulting in increased differentiation of NB cells. Patient data showed an inverse correlation between ARID1A and TERT expression, indicating a putative tumor suppressor role for ARID1A in NB [194].

These studies highlight that mutations in members of chromatin remodeling complexes are a feature of NB. The next step would be to elucidate the changes at any given nucleosome and associated gene expression, which will provide a strong base of knowledge in directing new avenues in epigenetic cancer therapies.

## Conclusions

To date, intensive efforts have been made to identify biomarkers that are affected by the changes in the epigenome in NB. However, there is still no universal, effective epigenetic biomarker allowing for the diagnosis and prognosis of NB. Thus, lack of molecular biomarkers for early detection of the disease remains one of the major hurdles in NB diagnosis, treatment, and prognosis. Therefore, a clinically effective approach would be an unbiased take between control and NB, regardless of their genetic background based solely on epigenetic alterations. Additionally, it is pivotal to identify changes in the methylome at single base resolution as opposed to entire genic regions, which is the current approach. This would allow for quick and efficient targeted testing in clinical settings.
